# How photosynthetic performance impacts agricultural productivity in hybrid cotton offspring

**DOI:** 10.1016/j.heliyon.2024.e34603

**Published:** 2024-07-14

**Authors:** Zexing Zhang, Hongliang Xin, Tianqi Jiao, Zhenhai Zhang, Ping He, Zhihui Yang, Jianbo Zhu, Ruina Liu

**Affiliations:** College of Life Sciences, Shihezi University, Shihezi, 832000, China

**Keywords:** Cotton, Crossbreeding, F2 generation, OJIP, Photosynthesis

## Abstract

Currently, heterosis is an effective method for achieving high crop quality and yield worldwide. Owing to the challenges of breeding and the high cost of the F1 generation, the F2 generation is considered the more desirable hybrid offspring for agricultural production. The use of OJIP fluorescence provides rapid insights into various photosynthetic mechanisms. However, OJIP fluorescence has not been previously studied as an indicator of the rate of heterosis. Consequently, we investigated the relationship between photosynthetic characteristics and growth and developmental parameters in hybrid cotton cultivars. The findings showed a gradual decline in the photosynthetic performance of hybrid cotton as the number of generations increased. In comparison to the F3 generation, both the F1 and F2 generations showed minimal variations in parameters, thus maintaining hybrid dominant and emphasizing the agricultural production potential of the F2 generation. The JIP-test revealed significant differences in the relationship between *ψ*_*Eo*_ and *ϕ*_*Eo*_ parameters, as well as variations in the connections between the photo-response center and electron transfer efficiency, and between cotton yield and fiber quality in the hybrid progeny. These variations can serve as indicators for predicting the extent of hybrid dominance in cotton. The results indicated significant differences in the light and dark responses of the hybrid offspring. By using parents with similar photosynthetic performance as genetic resources for crossbreeding, the photosynthetic capacity of the hybrid progeny can be enhanced to facilitate the efficient absorption and conversion of light energy in crops.

## 1 Introduction

Cotton is a widely grown cash crop worldwide, with significant economic and practical application potential [[1], [2], [3]]. The increasing demand for cotton fiber has made it urgent to improve cotton yield and quality [4], leading to the cultivation of high-quality and high-yield cotton varieties. Crop hybridization is one of the most effective methods for achieving high yields, superior quality, and increased stress resistance [[5], [6], [7], [8]]. The first generation (F1) of cotton hybrids offers a significant hybrid advantage, increasing yield by 20 % compared to conventional breeding. However, the seed production process requires a substantial amount of manpower and is expensive [9]. The second generation (F2) of hybrids exhibits 10 % hybrid dominance, which can be fully exploited to maximize hybrid dominance and thus meet human needs [10,11]. Breeders have proposed various concepts regarding the maintenance of hybrid dominance in the third generation (F3) of the cross. Vogel and Mitchell reported higher yields in the F3 generation than in F2 generation of *Panicum virgatum* L. [12]. Song et al. discovered that in rice, the hybrid advantage of the F1 generation was significantly greater than that of the F2 and F3 generations. Nevertheless, the agronomic traits of the F2 and F3 generations did not differ significantly from those of the parents [13]. Furthermore, the performance of hybrid Sea Island cotton remained consistent across both the F2 and F3 generations, as well as across various locations within the same generation. However, the prevalence of mesophilic dominance in hybrids between the F2 and F3 generations did not show any significant relevance [14]. Karademir. et al. proposed that in fiber fineness F2 generations hybrids were slightly higher than average of parental genotypes, but F1 generations hybrids were lower than both F2 generations and parent [15].

Crop hybrids are crucial cotton breeding, which makes it necessary to explore accelerated techniques for evaluating superior hybrid offspring. Establishing an efficient production system using light energy constitutes the primary goal of crop cultivation, highlighting the importance of improving crop light energy utilization to increase crop yield [16,17]. Kautsky and Hirsh observed that dark-adapted photosynthetic organs rapidly increased in chlorophyll fluorescence after being exposed to light, gradually declined, and eventually reached a stable value. This led them to realize the close relationship between the photosynthetic primary response and chlorophyll fluorescence(Chl F) [18]. As the study progressed, Chl F measurements were found to serve as a unique benchmark for enhancing global agricultural productivity models and improving crop yield predictions in the face of climate change [19]. Chl F technology is increasingly emerging as a potent tool in agricultural, environmental, and ecological research [20]. The JIP-test is a method for analyzing and processing fast chlorophyll fluorescence induction curves based on bioenergy flow. This test provides a robust and convenient method to investigate the initial reaction of photosynthesis by allowing analysis of fluorescence changes occurring in less than 1 s. It serves as an effective and convenient tool for in-depth examination of the primary response to photosynthesis. Such analyses provide comprehensive insights into the status and functionality of PSII reaction centers and antenna systems, as well as the donor and acceptor sides of PSII [21]. As a result, numerous studies have used Chl F for many years to measure plant photosynthetic responses under various abiotic stressors, such as drought and water scarcity [22,23], nutrient deficiencies [24,25], temperature fluctuations [[26], [27], [28]], radiation exposure [29], and others [27,30,31].

The photosynthetic process influenced the biomass and yield of photosynthetic organisms. The photochemical response is essential for efficient photosynthesis. The use of hybrid technology significantly increases crop yield. Many agriculturists prefer the F2 generation. OJIP fluorescence parameters are frequently used to assess the photochemical response of plants to abiotic stress. However, their variation in the offspring of crosses is not well understood. To investigate this, we selected three consecutive generations of cotton hybrids as experimental materials for statistical analysis. The objective of this study was to thoroughly evaluate potential productivity variations between generations of hybrid cotton using the OJIP fluorescence parameters, growth parameters, and agronomic traits of hybrid cotton offspring. Additionally, our aim was to demonstrate the feasibility of using OJIP fluorescence as a tool for assessing crop hybrid dominance rates. Providing a reference point for growth regulation approaches in cotton.

## 2 Results

### 2.1 Analysis of gas exchange data

**Fig. 1:**
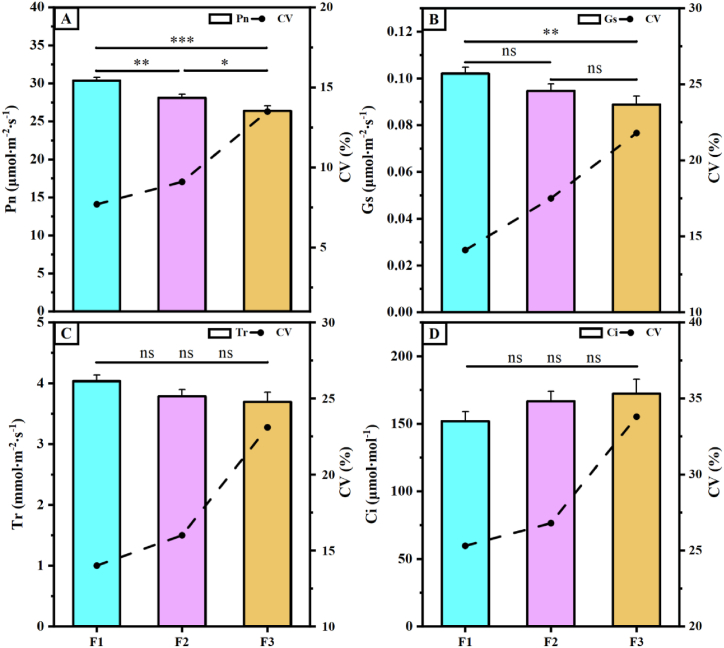
Gas exchange parameters of hybrid cotton offspring. A, Pn is net photosynthetic rate; **B**, Gs is stomatal conductance; **C**, Tr is transpiration rate; **D**, Ci is intercellular carbon dioxide concentration. CV is coefficient of variation. F1, F2, and F3 indicate three hybrid cotton offspring, ** indicates p ≤ 0.01, and *** indicates p ≤ 0.001, “ns”indicates that there is no significant difference.

In the study of plant growth and development processes, gas exchange parameters are influential factors in characterizing the photosynthetic efficiency of plants. Upon examination of the gas exchange parameters (Fig. 1), highly significant differences were observed in Pn among the offspring, with F1 exhibiting the highest Pn value (30.37 μmol m^−2^ s^−1^), which was 7.42 and 13.11 % higher than that of F2 and F3, respectively(Fig. 1A). Similarly, Tr of F1 was greater than that of F2 and F3, with Tr values of 4.03, 3.78, and 3.69 μmol m^−2^ s^−1^ for F1, F2, and F3, respectively(Fig. 1C). Although there were variations in Tr, Gs, and Ci among the offspring, these differences were not statistically significant(Fig. 1B–D). The F2 generation exhibited lower coefficients of variation (CV) for gas exchange parameters than the F1 and F3 generations.

### 2.2 Differences in key genes of the Calvin cycle

**Fig. 2:**
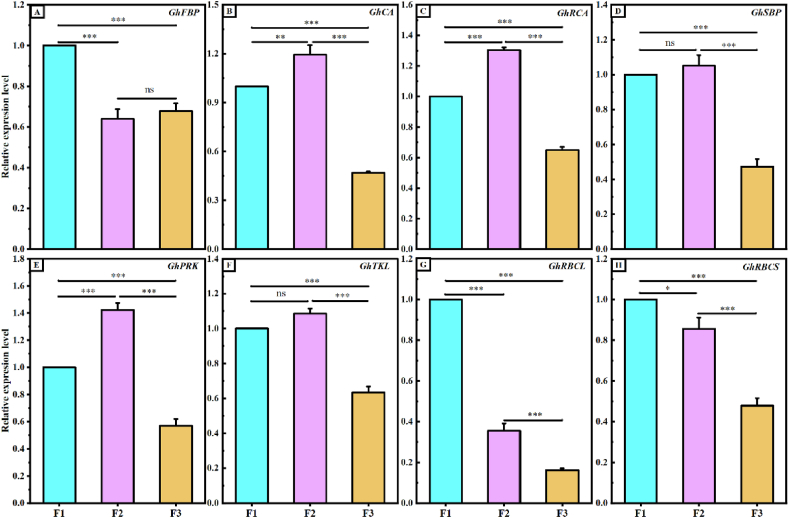
Relative expression levels of key enzyme genes of the Calvin cycle among hybrid cotton progeny. A, *GhFBP* is the cotton fructose bisphosphatase gene; **B**, *GhCA* is the carbonic anhydrase gene in cotton; **C**, *GhRCA* is the activase gene of ribulose bisphosphate carboxylase/oxygenase in cotton; **D**, *GhSBP* is the gene for cotton Sedum heptoketone dihydrogen diphosphatase; **E**, *GhPRK* is the riboketone phosphate kinase gene in cotton; **F**, *GhTKL* is the transketolase gene in cotton; **G** and **H**, *GhRBCL* and *GhRBCS* are the large and small subunit genes of ribulose bisphosphatase. F1, F2, and F3 indicate the three cotton offspring of the crosses, and * indicates p ≤ 0.05, ** indicates p ≤ 0.01, and *** indicates p ≤ 0.001, “ns” indicates no significant difference.

To further investigate the variations in photosynthesis among offspring, this study examined the gene expression levels of key enzymes involved in the Calvin cycle and associated with photosynthetic metabolites. The findings revealed (Fig. 2) that the relative expressions of the *GhRCA*, *GhCA*, and *GhPRK* genes were significantly higher in F2 than in F1 and F3(Fig. 2B,C,E). However, the relative expressions of the *GhTKL* and *GhSBP* genes were significantly higher in F2 than in F3, while no significant difference was observed between F2 and F1(Fig. 2D–F). Moreover, the relative expressions of the *GhFBP*, *GhRBCL*, and *GhRBCS* genes were significantly higher in F1 than in F2 and F3(Fig. 2A–G,H), whereas an overall lower expression was detected for Calvin cycle-related enzyme genes in general.

### 2.3 Chlorophyll fluorescence kinetic curve changes

**Fig. 3:**
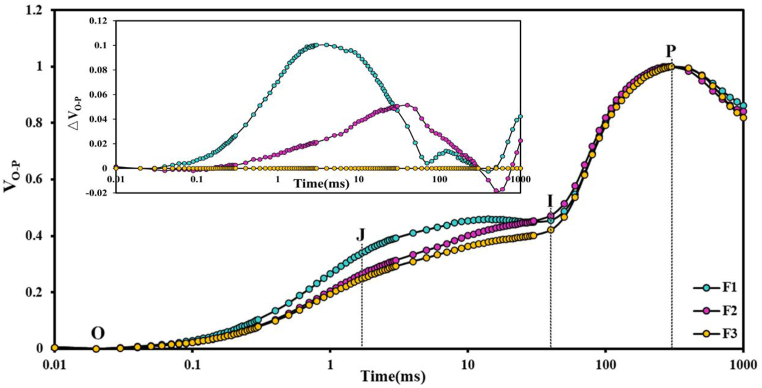
**Standardised chlorophyll fluorescence kinetic curves [V**_**O-P**_ = **(Ft-Fo)/(Fm-Fo)] and relative differences in standardised fluorescence intensities [ΔVO-P]VO-P (hybrid cotton progeny) - VO-P (hybrid cotton F3 generation)] for leaves of hybrid cotton progeny.** Where O is the chlorophyll fluorescence intensity at 20 μs, J is the chlorophyll fluorescence intensity at 20 ms, I is the chlorophyll fluorescence intensity at 30 ms, and P is the maximum fluorescence intensity. F1, F2, and F3 indicate the three hybrid cotton offspring.

The fast chlorophyll fluorescence kinetic curve (OJIP), which reflects the electron transfer and activity characteristics of the photochemical system, accurately represents the redox state of the PSII donor side, PSII acceptor side, and reaction center electrons in the light reaction. As illustrated in Fig. 3, the progeny exhibited an increasing trend with logarithmic time. The F1 generation was higher than the F2 and F3 generations in the J and I phases and lower than the F2 generation between the I and P phases. Throughout the entire process, F3 consistently remained lower than F1 and F2. In comparison to the F3 generation, the F1 and F2 generations exhibited predominantly positive ΔV_O-P_ values.

### 2.4 Changes in the photosynthetic electron transport chain

To examine the variations in the electron transport chain of the photosynthetic system among offspring, we employed JIP-test parameters to assess alterations in electron transport chain activity (Table 1). Significant differences were observed in the quantum efficiency and performance parameters (*Fv/Fm*, *Fv/Fo*, *PI*_*ABC*_, *DF*_*ABC*_, *ψ*_*Eo*_, *ϕ*_*Po*_, *ϕ*_*Eo*_, *ϕ*_*Ro*_, *δ*_*Ro*_) among offspring, while the structural indicators (*ABC/RC*, *TRo/RC*, *ETo/RC*, *REo/RC*) did not exhibit significant variations. For example, the F3 generation exhibited the highest potential photosynthetic efficiency (*Fv/Fo*) with a mean value of 3.98, whereas the F1 generation demonstrated the lowest Fv/Fo (3.38). In a similar way, the highest photosynthetic performance index (*PI*_*ABC*_) was recorded in the F3 generation (8.61), and the lowest was recorded in the F1 generation (5.05), but not in the F2 generation. The drive for photosynthesis (*DF*_*ABC*_) in the F1 generation was 20.02 % lower than that of the F2 generation and 24.69 % lower than that of the F3 generation. The variation in quantum efficiency was evident in the energy capture quantum efficiency (*ϕ*_*Po*_), which was lowest (0.77) in the F1 generation and highest (0.80) in the F3 generation, showing an increase with each subsequent generation. The quantum efficiency of intersystem electron transfer (*ϕ*_*Eo*_) was highest in the F3 generation (0.58), lowest in the F1 generation (0.50), and intermediate in the F2 generation (0.57). *ψ*_*Eo*_ followed the same trend as *ϕ*_*Po*_ and *ϕ*_*Eo*_, with values of 0.65, 0.71, and 0.73 for the F1, F2, and F3 generations, respectively. The quantum efficiency of PSI-terminal electron acceptor reduction (*ϕ*_*Ro*_) was highest between the F1 and F2 generations, with no significant difference (0.43), but these values were significantly lower than that in the F3 generation. *δ*_*Ro*_ was highest in the F1 generation (0.87) and showed values of 0.76 and 0.82 in the F2 and F3 generations, respectively. In contrast, the differences in the JIP-test parameters of the F1 generation were more pronounced between the F2 and F3 generations. However, between the F2 and F3 generations, only the values of *ϕ*_*Ro*_ were significantly different. The CV appeared irregular, possibly due to an insufficient sample size.

**Table 1:** **Statistics of chlorophyll fluorescence parameters of hybrid cotton offspring.** Values are means ± SD. Means within a column followed by a different letter are significantly different (P < 0.05). CV_1_, CV_2_ and CV_3_ is the coefficient of variation of each cross progeny.

OJIP-test	F1	CV_1_(%)	F2	CV_2_(%)	F3	CV_3_(%)
Structural Indicators & Fluxes						
*ABS/RC*	1.33 ± 0.0212 a	5.0	1.30 ± 0.0337 a	8.2	1.30 ± 0.0370 a	9.0
*DIo/RC*	0.31 ± 0.0092 a	9.5	0.27 ± 0.0090 b	10.4	0.26 ± 0.0107 b	12.8
*TRo/RC*	1.03 ± 0.0161 a	5.0	1.02 ± 0.0258 a	8.0	1.04 ± 0.0289 a	8.8
*ETo/RC*	0.66 ± 0.0327 a	15.6	0.73 ± 0.0309 a	13.3	0.76 ± 0.0299 a	12.4
*REo/RC*	0.57 ± 0.0214 a	11.8	0.56 ± 0.0342 a	19.2	0.62 ± 0.0304 a	15.4
Quantum Efficiencies						
*ϕ*_*Po*_	0.77 ± 0.0051 b	2.1	0.79 ± 0.0031 a	1.2	0.80 ± 0.0050 a	2.0
*ψ*_*Eo*_	0.65 ± 0.0264 b	12.9	0.71 ± 0.0126 a	5.6	0.73 ± 0.0104 a	4.5
*ϕ*_*Eo*_	0.50 ± 0.0226 b	14.4	0.57 ± 0.0100 a	5.6	0.58 ± 0.0109 a	5.9
*δ*_*Ro*_	0.87 ± 0.0382 a	13.8	0.76 ± 0.0186 b	7.7	0.82 ± 0.0160 ab	6.2
*ϕ*_*Ro*_	0.43 ± 0.0126 b	9.3	0.43 ± 0.0149 b	10.9	0.48 ± 0.0107 a	7.1
Performance Index and Driving Forces						
*Fv/Fm*	0.77 ± 0.0050 b	2.1	0.79 ± 0.0031 a	1.20	0.80 ± 0.0050 a	2.0
*Fv/Fo*	3.38 ± 0.0980 b	9.2	3.79 ± 0.0724 a	6.00	3.98 ± 0.1270 a	10.1
*PI*_*ABC*_	5.05 ± 0.6227 b	39.0	7.51 ± 0.4439 a	18.7	8.61 ± 0.6894 a	25.3
*DF*_*ABC*_	0.70 ± 0.0497 b	22.6	0.87 ± 0.0236 a	8.6	0.92 ± 0.0323 a	11.1

### 2.5 Variation in pigment content

**Fig. 4:**
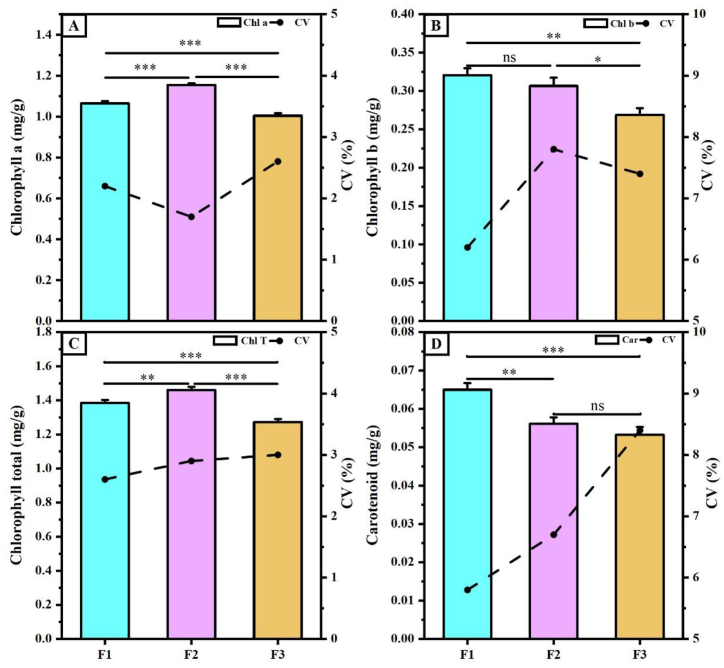
**Leaf pigmentation content of hybrid cotton progeny**. **A**, Chl a is chlorophyll *a* content; **B**, Chl b is chlorophyll *b* content; **C**, Chl T is chlorophyll content; **D**, Car is carotenoid content. CV is coefficient of variation, where F1, F2, and F3 indicate three hybrid cotton progeny, and * indicates p ≤ 0.05, “ns” indicates no significant difference.

Fig. 4 shows the pigment content (chlorophyll *a*, chlorophyll *b*, chlorophyll T,and carotenoids) of each cross progeny. The variation in pigment content among the offspring was considerable. The F2 generation of the cross had the highest chlorophyll *a*(Chl a) content, with a mean value of 1.15 mg g^−1^, which was 8.40 and 12.96 % higher than the F1 and F3 generations of the cross, respectively (Fig. 4A). Similarly, chlorophyll T(Chl T) showed the highest content in the F2 generation of the cross, which was 5.44 and 12.83 % higher than in the F1 and F3 generations, respectively(Fig. 4C). Similarly, the chlorophyll *b*(Chl b) content was higher in the F1 generation (0.32 mg g^−1^) than in the F2 (0.31 mg g^−1^) and F3 generations (0.27 mg g^−1^) (Fig. 4B). The carotenoid(Car) content was highest in the F1 generation (0.065 mg g^−1^) and lowest in the F3 generation (0.053 mg g^−1^) (Fig. 4D). Overall, the CV data showed a trend of F1 < F2 < F3.

### 2.6 Agronomic trait differences

**Fig. 5:**
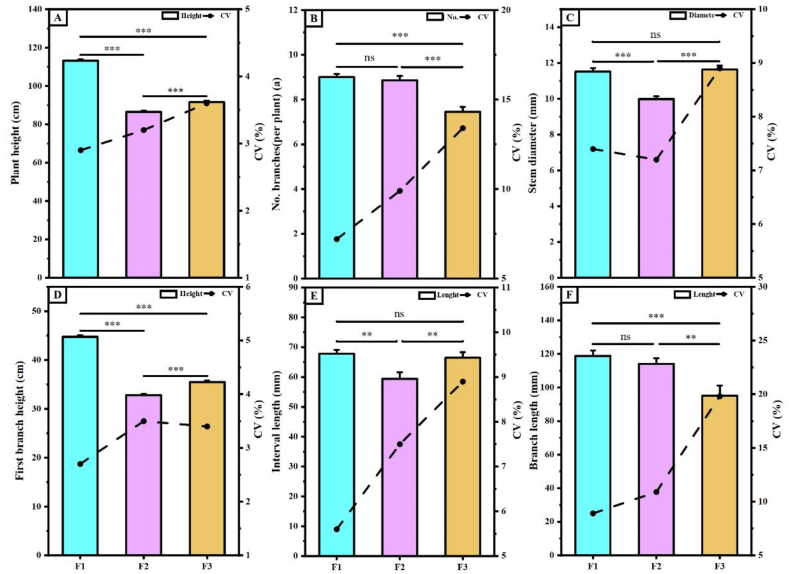
**Agronomic traits of hybrid cotton progeny. A**, plant height; **B**, Number of branches per plant; **C**, stem diameter; **D**, first branch height; **E**, interval length; **F**, branch length. CV is coefficient of variation. Where, F1, F2, F3 indicate three hybrid cotton progeny, * indicates p ≤ 0.05, ** indicates p ≤ 0.01, *** indicates p ≤ 0.001, “ns” indicates no significant difference。.

As shown in Fig. 5, the agronomic traits of each offspring varied significantly among the offspring under the same growing conditions. For example, F1 exhibited a greater plant height of 113.22 cm, which was 23.66 % higher than F2 and 19.14 % higher than F3, and a first fruiting branch height of 44.77 cm, which was 26.75 and 20.73 % higher than F2 and F3, respectively(Fig. 5A–D). The number of branches per plant was comparable between F1 and F2 at 9.00 and 8.85, respectively(Fig. 5B). The stem diameter significantly higher in F1 and F3 than in F2, at 11.51, 9.98, and 11.63 mm, respectively(Fig. 5C). The internode length of F2 was 10.57–12.44 % shorter than that of F1 and F3(Fig. 5E). The fruiting branch lengths were similar in the F1 and F2 generations, with the shortest boll length (95.07 mm) in the F3 generation(Fig. 5F).

**Fig. 6:**
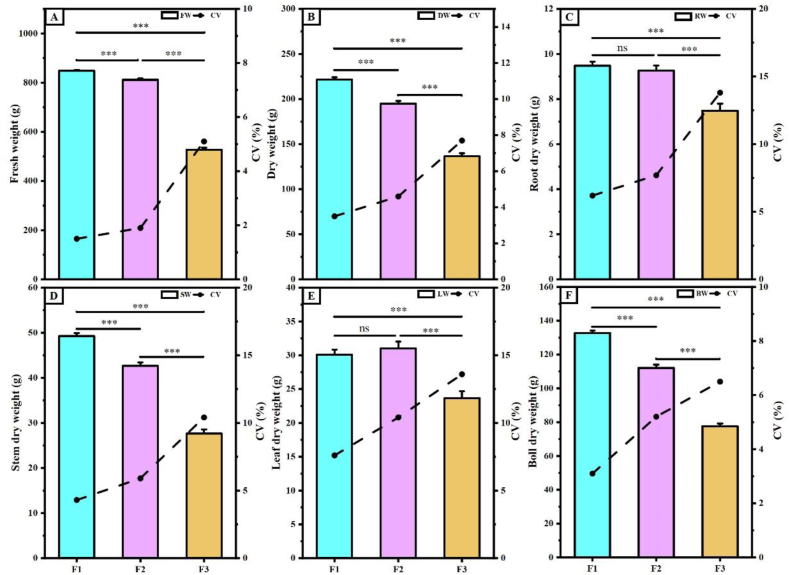
**Amount of dry matter in single plants and various tissues of hybrid cotton progeny. A**, fresh weight; **B**, dry weight; **C**, root dry weight; **D**, stem dry weight; **E**, leaves dry weight; **F**, boll dry weight. CV is coefficient of variation. Where F1, F2 and F3 indicate three hybrid cotton offspring, * indicates p ≤ 0.05, ** indicates p ≤ 0.01 and *** indicates p ≤ 0.001, “ns” indicates no significant difference.

As shown in Fig. 6, there was a significant difference in dry matter accumulation and fresh weight among the progeny. F1 exhibited the highest dry matter accumulation (221.55 g·plant^−1^), followed by F2 (194.95 g·plant^−1^) and F3 (136.41 g·plant^−1^) (Fig. 6B). Fresh weight exhibits similar behaviour to its(Fig. 6A). There were differences in the allocation of dry matter among various tissues (roots, stems, leaves, and bolls) in the offspring(Fig. 6C–F). Notably, among the tissues, the reproductive organ cotton bolls were 15.59 and 41.49 % heavier in F1 than in F2 and F3, respectively. The roots and stems exhibited a similar pattern. However, F2 had the highest dry matter weight of leaves, at 31.01 g·plant^−1^. The dry matter mass of the roots was comparable between the F1 and F2 generations, while the lowest root weight was found in the F3 generation (7.48 g·plant^−1^). In addition, the fresh weight of F1 was significantly higher than that of F2 and F3, by 4.33 and 37.86 %, respectively. The F2 generation had lower CVs for agronomic traits than the F3 generation, relative to the F1 generation.

### 2.7 Yield trait and fiber quality differences

**Fig. 7:**
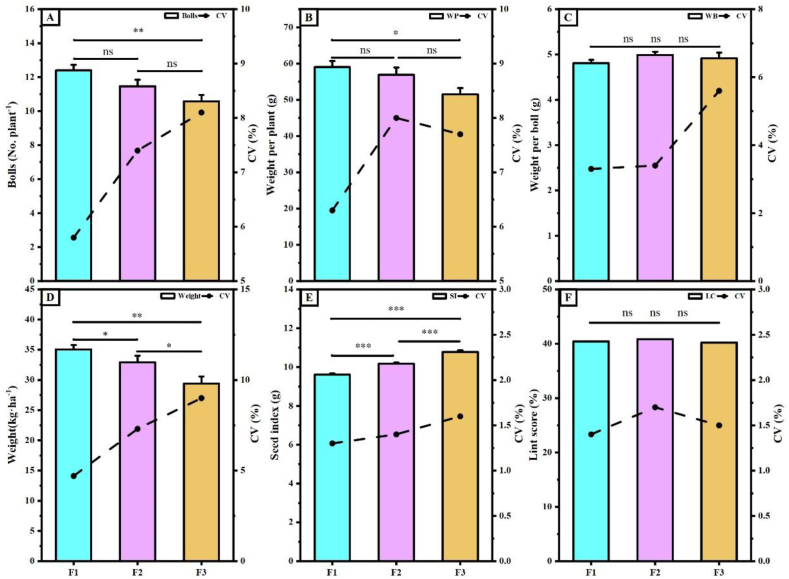
**Yield traits of hybrid cotton progeny**. **A**, Number of bolls per plant; **B**, Weight of seed cotton per plant; **C**, Weight of a single boll; **D**, Seed cotton weight per hectare; **E**, seed index; **F**, lint score. CV is coefficient of variation, where F1, F2, and F3 indicate three hybrid cotton progeny, * indicates p ≤ 0.05, ** indicates p ≤ 0.01, and *** indicates p ≤ 0.001, “ns” indicates no significant difference.

The yield traits of the hybrid cotton progeny are illustrated in Fig. 7. The parameters of the seed index differed significantly among the progeny. The F1 generation had the smallest seed index (9.61 g), which was 5.54 and 10.82 % smaller than the F2 and F3 generations, respectively(Fig. 7E). The yield trait parameters differed among generations, with the highest values observed in the F1 generation at 35.06 kg ha^−1^, which was 2.13 and 3.55 kg ha^−1^ more than that of the F2 and F3 generations, respectively(Fig. 7D). The F1 and F3 generations showed significant differences in the number of bolls and seed cotton weight per plant(Fig. 7A and B). The boll weight per boll and lint score did not differ significantly among the offspring(Fig. 7C–F). However, the CV for each yield trait parameter increased with the number of passages in hybrid cotton during outgoing passages.

**Fig. 8:**
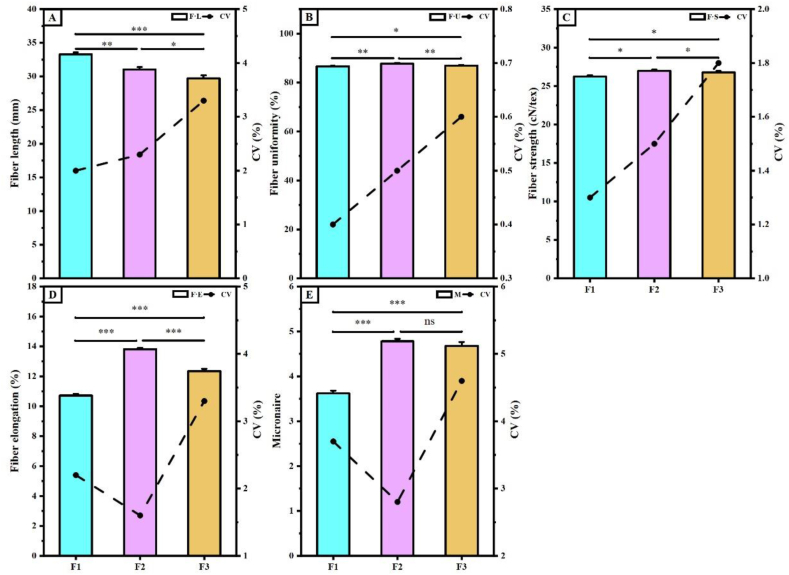
Comparison of differences in fiber quality among hybrid cotton progeny. A, fiber length; **B**, fiber uniformity; **C**, fiber strength; **D**, fiber elongation; **E**, micronaire. CV is coefficient of variation, where F1, F2, and F3 indicate three hybrid cotton progeny, * indicates p ≤ 0.05, ** indicates p ≤ 0.01, and *** indicates p ≤ 0.001, “ns” indicates no significant difference.

Fig. 8 shows that the fiber quality of hybrid cotton progeny varied significantly among generations, with the CV increasing with the number of passages. Specifically, the F·L of the F1 generation was 7.22 % longer than that of the F2 generation and 11.98 % longer than that of the F3 generation. The CVs were 2.00 %, 2.30 %, and 3.30 %, respectively (Fig. 8A). The F2 generation exhibited the highest values for F·U, F·S, F·E, and M, which were significantly greater than those of the F1 and F3 generations. Additionally, the coefficient of variation showed an increasing trend (Fig. 8B–E).

**Fig. 9:**
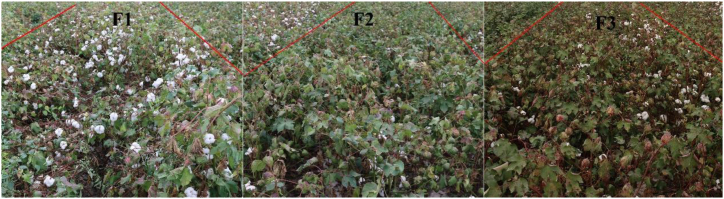
**Hybrid cotton offspring field planting and growth**, where F1, F2, and F3 denote three hybrid cotton progeny.

The growth of hybrid cotton offspring in the field is depicted in Fig. 9. In particular, the F1 generation exhibited greater plant height and earlier maturity (complete fluffing by September 25) but was prone to collapse during the boll stage. In contrast, the F2 generation had a shorter plant height and did not topple over but reached maturity slightly later (completed fluffing on October 5). The F3 generation exhibited a plant height similar to that of the F2 generation, and its maturity fell between that of the F1 and F2 generations (completed fluffing on October 1). However, there was a significant difference in plant shape.

### 2.8 Pearson's correlation analysis

**Fig. 10:**
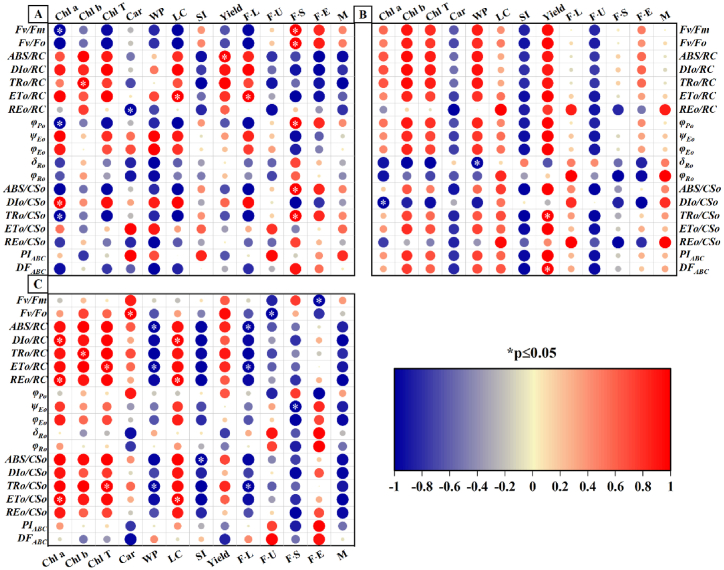
**Correlation analysis between pigment content, yield traits and fiber quality of hybrid cotton offspring and JIP-parameters.** Where A is the correlation in F1 generation, B is the correlation in F2 generation and C is the correlation in F3 generation. Red colour in all graphs indicates positive correlation, blue colour indicates negative correlation, and correlation coefficient above 0.7 indicates very strong relationship, between 0.4 and 0.7 indicates strong relationship, 0.2 and 0.4 indicates fair relationship, and 0 to 0.2 indicates no correlation. LC, SI, WP, Yield, F·L, F·U, F·S, F·E and M denote indicate lint score, seed index, weight per plant, yield, fiber length, uniformity, strength, elongation, and micronaire respectively. * indicates highly significant at p ≤ 0.05. (For interpretation of the references to colour in this figure legend, the reader is referred to the Web version of this article.)

To gain a deeper understanding of the potential relationship between pigment content, yield traits, and fibre quality with JIP parameters, Pearson correlation analysis was conducted for each parameter in F1, F2, and F3. As shown in Fig. 10A, positive correlations (r = 0.43–1.00) were observed between chlorophyll content and *ABS/RC*, *DIo/RC*, *TRo/RC*, *ETo/RC*, *ψ*_*Eo*_, *ϕ*_*Eo*_, and *DIo/CSo* of F1 generation. Similarly, lint score(LC), yield, and fiber length(F·L) exhibited similar correlations with the pigment content(r = 0.37–1.00). In addition, the F·S, F·E and M of the fibers showed similar positive correlations with *Fv/Fm*, *Fv/Fo*, *ϕ*_*Po*_, *ABC/CSo*, and *TRo/CSo*(r = 0.44–1.00).

There were more significant correlations between all trait parameters, except for Car, SI and F·U, with JIP parameters in the F2 generation (Fig. 10B). Notably, the chlorophyll content was largely positively correlated (r = 0.26–0.96) with all other parameters, except for a low correlation with *REo/RC*, *ϕ*_*Ro*_, *δ*_*Ro*_, *DIo/CSo*, and *REo/CSo*. Strong positive correlations were observed between WP and yield, with nearly all JIP parameters in yield traits (r = 0.43–1.00). SI was positively correlated with *δ*_*Ro*_ (r = 0.49). In addition, The correlation between F·L and M with JIP parameters was almost identical, the variables with the highest correlation are *REo/RC*(r = 0.87), *ϕ*_*Ro*_(r = 0.98), *DIo/CSo*(r = 0.82), and *REo/CSo* (r = 1.00).

Fig. 10C shows a higher correlation between chlorophyll and JIP parameters in the F3 generation, similar to the F2 generation. However, the performance of Car, WP, F·L, and M in the F3 generation is opposite to that in the F2 generation. Yield traits SI and WP displayed negative associations with the JIP-test. The correlation coefficients between pigment content and energy absorption, as well as transfer in the reaction center, ranged from 0.32 to 1.00, while those with energy absorption and conversion in the initial light-receiving cross section ranged from 0.29 to 1.00. Moreover, there was a strong positive correlation (r = 0.89–1.00) between LC and energy flow in both the photosynthetic reaction center and the initial light cross sections. In terms of fiber quality, F·U showed positive correlation with *ϕ*_*Ro*_, *δ*_*Ro*_, *PI*_*ABC*_ and *DF*_*ABC*_.F·S showed positive correlation only with *Fv/Fm* (*ϕ*_*Po*_), r = 0.74. F·E showed positive correlation with quantum efficiencies or flux ratios and initial apparent quantum flux per unit light-exposed cross-sectional area.

## 3 Discussion

The findings of this study demonstrated that the F2 generation exhibited a higher potential for application and research value in agricultural production, aligning with previous studies [6,7,32,33]. The study revealed a significant variation in photosynthetic performance among the offspring, and the F1 generation exhibited a higher photosynthetic capacity than the F2 and F3 generations. The same variability was observed in agronomic and yield traits. Nevertheless, previous studies have underutilized the relationship between chlorophyll fluorescence kinetic parameters and other parameters in explaining the differences that arise in hybrid cotton during successive passages.

Currently, cross-breeding is widely employed as the primary approach in cotton breeding [34]. The study showed that F1 and F2 generation hybrid cotton had a significant growth advantage over the parents [5,35,36]. Among the agronomic traits observed in the progeny from the three crosses in this study, the plant height, first fruiting branch height, stem thickness, and internode length of the F2 generation were significantly lower than those of the F1 and F3 generations. The number of fruiting branches in the F1 generation was greater than that of the F2 and F3 generations, and the difference in the F2 generation was smaller (Fig. 5). The plant's fresh weight and dry matter accumulation of the F1 generation were higher than those of the F2 and F3 generations. Additionally, the CVs of the F1 and F2 generations were smaller than those of the F3 generation (Fig. 6), indicating that the coefficient of variation of the offspring gradually increased with the number of passages. In terms of dry matter accumulation, the CV between the F2 and F1 generations was lower than that between the F3 and F1 generations. The formation of this difference may be related to the accumulation of dry matter in the source organs (Fig. 6). Yield traits are the most direct parameters for assessing crop yield [37,38]. The lint score of F2 generation was higher than that of the F1 and F3 generations. The F1 generation exhibited a clear advantage in terms of the number of bolls per plant, the weight of seed cotton per plant, and overall yield. However, compared to the F1 generation, the F2 generation showed good growth performance in the field, as did the F3 generation. Notably, the F1 generation experienced plant collapse, posed challenges for cotton harvesting (Fig. 8). Therefore, considering growth, development, and agricultural production potential, F2 generation hybrid cotton is especially suitable for practical production applications.

Gas exchange parameters are influential factors in characterizing the photosynthetic efficiency of plants during the study of plant growth and development processes [39,40]. Gs is the main driver of changes in Ci, Tr, and Pn in plants [[41], [42], [43], [44]]. In this experiment, both Pn and Gs were significantly higher in the F1 generation than in the F2 and F3 generations. These findings indicate that the higher Gs observed in the F1 generation led to increased Tr and decreased Ci levels, resulting in significantly higher Pn and overall enhanced photosynthetic efficiency than that in the F2 and F3 generations. This aligns with previous research results [16]. Similarly, photosynthetic metabolites play a crucial role in influencing the net photosynthetic rate among photosynthetic organisms [[45], [46], [47]]. Our analysis of the expression of key enzyme genes involved in the Calvin cycle in the leaves of three generations of hybrid cotton showed that the relative expression of *GhFBP*, *GhRBCL,* and *GhRBCS* genes was higher in the F1 generation than in the F2 and F3 generations, and the relative expression of *GhRCA*, *GhCA*, *GhPRK*, *GhTKL*, and *GhSBP* genes was higher in the F2 generation than in the F1 and F3 generations (Fig. 2). Among these genes, *GhRCA* plays a regulatory role as an activator gene for Rubisco within the Calvin cycle, enabling maximum activation of Rubisco in plants [48]. *CA*, *SBP,* and *RBCS* have also been reported to promote the carboxylation of RuBP, a substrate of Rubisco, which promotes CO_2_ fixation capacity and increases plant Pn [[49], [50], [51]]. The F2 generation has been selected over the F1 and F3 generations because of the heightened expression of crucial enzyme genes within the Calvin cycle. This elevation has resulted in an augmentation of photosynthetic metabolites, impacting the accumulation of dry matter in roots and leaves, and diminishing the CV between the F2 and F1 generations.

OJIP was used to quantify the effects of abiotic stress conditions, such as temperature, soil water deficits, and nutrient levels [22,23,25,27,28,52], on the structure and function of photosynthetic organs in plants [[53], [54], [55], [56]]. To our knowledge, few researchers have used OJIP curves and their derived parameters to analyze variations in photosynthetic processes among the offspring of hybrid crops. In our study, the analysis of OJIP curves and derived parameters among hybrid cotton progeny revealed differences in their photosynthetic processes as they passed through hybrid cotton. In Fig. 3, from the standardized OJIP curves, we found differences in performance between the hybrid cotton progeny in the I–P segment. This phenomenon, most pronounced in the F2 generation and second most pronounced in the F1 and F3 generations, indicates that the F2 generation has the highest electron transfer efficiency from PQ to the end of PS I. ΔV_O-P_ was used to compare the extent of damage to the photosynthetic apparatus of plant leaves under abiotic stress conditions. We utilized it as a tool to assess alterations in the rate of hybrid dominance in hybrid offspring. The ΔV_O-P_ values of the F1 and F2 generations exhibited predominantly positive values in comparison to those of the F3 generation. ΔV_O-P_ can be used to objectively evaluate the photosynthetic capacity of hybrid progeny.

The JIP-test has primarily been used to analyze changes in the structure and function of plant photosynthetic organs under different environmental conditions [25,28,30]. In this paper, we focus on the analysis of the photoreaction centers of hybrid cotton passages regarding quantum efficiency, performance index, and driving force. Table 1 shows that *Fv/Fm*, *Fv/Fo*, *PI*_*ABC*_, *DF*_*ABC*_, *ψ*_*Eo*_, *ϕ*_*Po*_, *ϕ*_*Eo*_, *ϕ*_*Ro*_, and *δ*_*Ro*_ of the F1 generation were significantly lower than those of the other two generations. Among them, *Fv/Fo* is related to the donor-side activity of the PS II reaction center, and *ψ*_*Eo*_ and *ϕ*_*Eo*_ are related to the acceptor-side activity of the PS II reaction center. *Fv/Fm* and *PI*_*ABC*_ can characterize the photosynthetic performance of PS II [27,28]. Therefore, the activity of PS II is lower in the F1 generation, reducing the activity of the electron transport chain. This ultimately resulted in the lower effective photosynthetic efficiency in the F1 generation than in the F2 and F3 generations, leading to lower photosynthetic performance. The electron transfer process of the fast-reducing PQ pool decreased in the F3 generation at a rate comparable to that of the F1 generation. The F3 generation exhibited an increased capacity for primary photochemical reactions, potentially leading to the accumulation of active chemical energy and the generation of superoxide radicals and affecting light energy conversion processes, such as the Calvin cycle in plants [57]. Therefore, we suggest that the appropriate photochemical reaction activity may be responsible for the higher photosynthetic rates of the F1 and F2 generations.

Numerous studies have shown a correlation between pigment content, biomass accumulation, and the JIP-test [25,27,28]. In this study, we correlated pigment content, yield traits and fiber quality using the JIP-test. We observed a strong correlation between chlorophyll content and *ψ*_*Eo*_, *ϕ*_*Eo*_, and structural indicators, which are associated with the activities of the PS II reaction center on the receptor side, the quantum yields of light energy captured for Q_A_^−^ downstream electron transfer, and the energy absorbed for electron transfer in the PS II reaction center of the plant's photosynthetic organ. Hence, there is a positive association between chlorophyll content and the photosynthetic electron transfer capacity of the PS II reaction center complex. Compared with the F2 generation, the F1 and F3 generations exhibited a positive correlation between *DIo/CSo* and pigment content. This suggests that the thermal dissipation of energy does not affect pigment accumulation in the F2 generation with highly active PS II. Interestingly, the correlation between Car content and the JIP-test varied significantly among offspring, with all showing negative correlations in the F2 generation. However, in the F3 generation, there was a strong correlation between Car and *Fv/Fm* (*ϕ*_*Po*_) as well as *Fv/Fo*. Car is accessory pigments in the membranes of chloroplasts that aid in absorbing light that chloroplasts cannot absorb, thereby enhancing photosynthetic efficiency and offering crucial protection against light damage [58,59]. Therefore, we suggest that the reduction in Car content during hybrid cotton passages prevents superoxide radical formation. This is due to the rapid conversion of active chemical energy generated in the photosynthetic reaction center of the F2 and F3 generations. This leads to photodamage and affects the photosynthetic efficiency of plants.

Yield traits are directly related to crop productivity [37,38]. Correlations between the LC and JIP-tests were found in all hybrid cotton progeny, primarily in the PS II reaction center and electron transport processes. In the F1 generation, the yield was only correlated with parameters related to the photoreaction center. However, in the F2 and F3 generations, yield was positively correlated with almost all JIP parameters, indicating that crop yield may decrease with increasing photoreaction center activity. The yield per plant in the F1 and F2 generations was primarily associated with *ψ*_*Eo*_ and *ϕ*_*Eo*_. The lint scores were positively correlated with all parameters in the F2 generation, indicating a possible relationship between LC and the photosynthetic reaction center in the F2 generation. And the yield of the F2 generation also exhibited a strong correlation with *ψ*_*Eo*_ and *ϕ*_*Eo*_, whereas the yield of the F1 and F3 generations showed little correlation. In conclusion, the appropriate ranges of *ψ*_*Eo*_ and *ϕ*_*Eo*_ may be the key factors influencing yield traits in cotton.

Cotton fiber quality is a crucial factor in determining the quality of cotton textiles [60]. The selection of cotton varieties is determined by the fiber quality of hybrid cotton offspring. Our experiments revealed significant differences in the correlation between cotton fiber quality and the JIP-test. The correlation between F·L and photo-response centers was positive in the F1 generation but decreased or even became negative with an increase in the number of passages. Additionally, the F·E of the F2 generation exhibited a weaker positive correlation with photoreaction centers and electron transfer efficiency, whereas the F·E of the F3 generation demonstrated a stronger correlation with electron transfer efficiency. Therefore, it is speculated that the secondary metabolic pathways in cotton affect the F·L and F·E of cotton fibers [60]. The moderate photochemical activity of the F2 generation reduces the accumulation of photochemical radicals, which, in turn, affects the growth of cotton fibers through secondary metabolic pathways. The neatness and specific strength of cotton fibers are minimally affected by chlorophyll fluorescence.

## 4 Conclusions

In summary, the objective of this study was to clarify the factors contributing to the preference for the F2 generation as the more desirable hybrid progeny in agricultural production. And to explore the potential use of OJIP fluorescence as an indicator for assessing the degree of hybrid dominance. This was accomplished by investigating the influence of the transmission process on the growth, development, and photosynthetic performance of the offspring resulting from landrace cotton crosses. The results showed that the transmission process significantly affected the growth, yield, and photosynthetic performance of hybrid cotton. The F2 generation showed better growth, yield, and photosynthetic performance than the F3 generation. The difference between the F2 and F1 generations was lower than that of the F3 generation, and this enabled the F2 generation to meet the current requirements for agricultural production. By analyzing the relationship between pigment content, yield characteristics and fiber quality of hybrid cotton progeny and the parameters of the JIP-test, we found that the inheritance of *ψ*_*Eo*_ and *ϕ*_*Eo*_ had a clear pattern and was highly correlated with the productivity parameters of cotton. In conclusion, appropriate photochemical activity is the main determinant of hybrid dominance in the F2 generation, and OJIP fluorescence can be an important tool for assessing the degree of hybrid dominance. In addition, if two cultivars with similar photosynthetic properties are crossed as parents, the photosynthetic dominance of the hybrid progeny may be enhanced.

## 5 Methods and materials

### 5.1 Plant material and experimental design

The materials used in the experiments were hybrid seeds of the F1, F2, and F3 generations of two random varieties. The experimental seeds were provided by the Academy of Agricultural Sciences of KuiTun City, Xinjiang Autonomous Region, China. The field experiment was conducted in 2023 at the experimental site of Shihezi University, located in Shihezi City, Xinjiang (45°19′N, 86°03′E). The experiment was set up in plots with a length of 5 m, a width of 2.27 m, a row spacing of 76 cm, and a spacing of 10 cm between plants in each row, and there were five randomly distributed replications. The seeds were sown on May 15, 2023, using spot sowing. Field management included drip irrigation, watering, fertilization, and meperidine application as a growth regulator every 7–8 days. The supply of water, fertilizer, and growth regulators was stopped in September.

### 5.2 Gas exchange parameters

Gas exchange parameters were measured using a Li-6400TX (LI‐COR Inc., Lincoln, USA) portable photosynthesis analyzer. The Li-6400TX built-in artificial light source was set at an intensity of 1200 μmol m^−2^ s^−1^ and a CO_2_ concentration of 400 ppm from 10:00 to 12:00 a.m. The net photosynthesis rate (Pn), stomatal conductance (Gs), interstitial CO_2_ concentration (Ci), and transpiration rate (Tr) were measured in the inverted 4-leafed main stems (inverted to 3-leafed after topping). Ten cotton leaves were measured in each plot, and each leaf was measured three times. The average of the three measurements was taken as the final value.

### 5.3 OJIP fluorescence

After topping the cotton (August 10), OJIP fluorescence was measured in cotton plants of the F1, F2, and F3 generations. Five consecutive plants were selected from each plot, and the plants were dark-adapted with the main stem with 3 leaves inverted for 20 min in the morning from 10:00 to 12:00. The rapid chlorophyll fluorescence induction kinetic curves of the leaf blades (OJIP curves) were determined using the Handy PEA instrument. The LED light source of 3000 μmol m^−2^ s^−1^ and a detection time of 1 s were used for rapid fluorescence signal acquisition. To minimize leaf heterogeneity, three points were measured for each leaf, whereupon the average value was used as the final fast fluorescence data. The formulas and meanings of the OJIP fluorescence parameters and their derived parameters are provided in Table 2 [27,52,61].

**Table 2:** JIP-test parameters used for analysis of Chl a fluorescence transient OJIP.

Parameters	Formulas	Explanation the technical data
Technical fluorescence parameters		
*F*_*o*_	*F*_*20μs*_	Fluorescence intensity at 20 μs, when all PSII RCs are assumed to be open
*F*_*j*_	*F*_*2ms*_	Fluorescence intensity at J-step (at 2 ms)
*F*_*i*_	*F*_*30 ms*_	Fluorescence intensity at I-step (at 30 ms)
*F*_*v*_	*F*_*m*_*-F*_*o*_	Maximal variable fluorescence
*F*_*v*_*/F*_*o*_	*(F*_*m*_*-F*_*o*_*)/F*_*o*_	Contribution of the thylakoid reactions to *PI*_*ABS*_.
Structural Indicators & Fluxes		
*ABS/RC*	*M*_*o*_*(1/V*_*j*_*) (1/ϕ*_*Po*_*)*	The specific energy fluxes per RC for energy absorption
*TR*_*o*_*/RC*	*M*_*o*_*(1/V*_*j*_*)*	Trapped energy flux (leading to QA reduction) per RC
*ET*_*o*_*/RC*	*M*_*o*_*(1/V*_*j*_*) (1-V*_*j*_*)*	Electron transport flux (further than Q_A_^−^) per RC
*RE*_*o*_*/RC*	*M*_*o*_*(1/V*_*j*_*) (1-V*_*i*_*)*	Electron flux reducing end electron acceptors at the PSI acceptor side per RC
*DI*_*o*_*/RC*	*ABS/RC-TR*_*o*_*/RC*	Effective dissipation per active RC
Quantum efficiencies or flux ratios		
*F*_*v*_*/F*_*m*_*(ϕ*_*Po*_*)*	*[1-(F*_*o*_*/F*_*m*_*)]*	The maximal quantum yield of PSII photochemistry
*ϕ*_*Do*_	*1-F*_*v*_*/F*_*m*_	The quantum yield of energy dissipation
*ψ*_*Eo*_	*(1-V*_*j*_*)*	The probability that an RC-trapped exciton moves an electron into the electron transport chain outside Q_A_^−^
*ϕ*_*Eo*_	*[1-(F*_*o*_*/F*_*m*_*)] (1-V*_*j*_*)*	The quantum yield of electron transport
*δ*_*Ro*_	*RE*_*o*_*/ET*_*o*_*=(1-V*_*i*_*)/(1-V*_*j*_*)*	Probability that an electron is transported from the reduced PQ to the electron acceptor side of PSI
*ϕ*_*Ro*_	*[1-(F*_*o*_*-F*_*m*_*)] (1-V*_*i*_*)*	Quantum yield of electron transport from Q_A_^−^ to the PSI end electron acceptors
Initial apparent quantum flux per unit light-exposed cross-sectional area		
*ABS/CS*_*o*_	*F*_*o*_	Absorption per excited cross-section.
*DI*_*o*_*/CS*_*o*_	*(ABS/CS*_*o*_*)-(TR*_*o*_*/CS*_*o*_*)*	Heat dissipation per excited cross-section.
*TR*_*o*_*/CS*_*o*_	*ϕ*_*Po*_*F*_*o*_	Trapping per excited cross-section.
*ET*_*o*_*/CS*_*o*_	*ϕ*_*Po*_*ψ*_*o*_*F*_*o*_	Electron transport
*RE*_*o*_*/CS*_*o*_		Electron flux reducing end electron acceptors at the PSI acceptor side, per excited cross-section.
Performance indexes		
*PI*_*ABC*_	$\left(\frac{RC}{ABC}\right)\frac{\phi_{Po}}{1‐\phi_{Po}}\frac{\psi_{o}}{1-\psi_{o}}$	The performance index for the photochemical activity
Driving Forces		
*DF*_*ABC*_	Log *(PIABC)*	Driving force based on absorbed light energy

### 5.4 Real-time fluorescence quantitative PCR (RT-qPCR)

At the boll stage (10 August), fresh leaves were collected from the main stem of the plant with four inverted leaves (three inverted leaves after topping). The leaves were quickly placed into liquid nitrogen and then stored in a −80 °C low-temperature freezer upon returning to the laboratory. RNA was extracted from the leaves following the manufacturer's instructions, and reverse transcription was carried out using a reverse transcription kit to obtain the cDNA strand. The concentration of cDNA was measured using a micro-nucleic acid meter, and real-time fluorescence quantitative PCR was performed by diluting the cDNA concentration to the same level.

Based on the sequences of key regulatory genes related to photosynthesis in upland cotton stored in GenBank and the gene sequence of the internal reference gene *GhUBQ7*, specific primers were designed using Primer-BLAST in the NCBI database. Gene expression was detected using a Bio-Rad real-time fluorescence quantification instrument and a BioGlod SYBR qPCR Master Mix kit from BIOGENE. The reaction procedure involved 40 cycles of incubation at 95 °C for 30 s, followed by denaturation at 95 °C for 5 s, and annealing at 59 °C for 30 s for the generation of lysis curves. The results were used to calculate the relative expression of genes using the 2^−ΔΔCt^ method [62].

### 5.5 Chlorophyll content and plant traits

Chlorophyll content and agronomic traits were assessed in cotton at the boll stage on July 20. Healthy, actively growing plants were selected for the experiment. Twenty leaf discs, each with a 1-cm diameter, were obtained from the fourth leaf of the main stem (or the third leaf after topping). The leaf discs were weighed and then placed in 10 mL of 95 % ethanol for extraction through soaking. Once the leaf discs turned white, the absorbance of the extracts was measured at 470, 645, and 663 nm using a spectrophotometer to calculate the chlorophyll and carotenoid content.

We selected 10 consecutive and well-established plants for agronomic trait analysis. Next, plant height, fruit-bearing node height, number of fruiting branches, number of flowering bells, internode length, and length of fruiting branches were measured. The plants were removed intact from the soil, and their fresh weight was quickly measured. The tissues were then dried separately at 105 °C and further dried at 80 °C until a constant weight was achieved. The dry weight of each plant was recorded.

### 5.6 Yield traits and fiber quality

Yield and yield traits were measured at cotton maturity on October 15. In each plot, 10 consecutive fully matured cotton bolls were collected from the plants. They were individually weighed and tested indoors for the seed index, and the lint score was calculated. The lint index was then calculated based on the lint score and seed index. Additionally, 50 fully matured bolls from the middle of the plant were collected and weighed to determine the average boll weight. The yield was indicated by the total weight of the fully matured seed cotton in the entire plot.

20g of lint cotton from each single plant was sent to the Xinjiang Autonomous Region Research Institute of Agricultural Sciences (XARIAS) for testing fiber quality. The testing included measurements of fiber length (F·L), uniformity (F·U), strength (F·S), elongation (F·E), and micronaire (M). Five samples were sent for each hybrid offspring.

### 5.7 Statistical analysis

SPSS 26.0 was used to test the data for normality and chi-square. The data were analyzed using one-way analysis of variance (ANOVA), and the means were separated using a *t*-test at a significance level of 0.05. Data were graphically plotted using Origin 2021 Pro.

## Funding

This work was supported by the Corps Financial Science and Technology Programme Project (Project No. 2023CB008-27 and 2023AB006-02).

## Data availability statement

Conflict of interest the authors declare that the research was conducted in the absence of any commercial or financial relationships that could be construed as a potential conflict of interest. There is no data for this paper that is stored in any publicly available databases.

## CRediT authorship contribution statement

**Zexing Zhang:** Writing – review & editing, Writing – original draft, Visualization, Methodology, Investigation, Data curation, Conceptualization. **Hongliang Xin:** Writing – review & editing, Writing – original draft, Methodology, Investigation, Conceptualization. **Tianqi Jiao:** Writing – review & editing, Writing – original draft, Methodology. **Zhenhai Zhang:** Writing – review & editing, Writing – original draft, Methodology, Investigation. **Ping He:** Writing – review & editing, Writing – original draft, Validation, Investigation, Data curation. **Zhihui Yang:** Writing – review & editing, Writing – original draft, Investigation. **Jianbo Zhu:** Writing – review & editing, Writing – original draft, Supervision, Resources, Project administration, Funding acquisition, Formal analysis, Data curation, Conceptualization. **Ruina Liu:** Writing – review & editing, Writing – original draft, Supervision, Resources, Project administration, Funding acquisition, Formal analysis, Data curation, Conceptualization.

## Declaration of competing interest

The authors declare that they have no known competing financial interests or personal relationships that could have appeared to influence the work reported in this paper.

## References

[1] Zhou M. (2014). Cotton proteomics for deciphering the mechanism of environment stress response and fiber development. J. Proteonomics.

[2] Jan M. (2022). Molecular regulation of cotton fiber development: a review. Int. J. Mol. Sci..

[3] Yang Z. (2022). Recent progression and future perspectives in cotton genomic breeding. J. Integr. Plant Biol..

[4] Hinze L.L. (2011). Performance and combining ability in cotton (Gossypium hirsutum L.) populations with diverse parents. Euphytica.

[5] McCarty J.C. (2007). Use of primitive derived cotton accessions for agronomic and fiber traits improvement: variance components and genetic effects. Crop Sci..

[6] Campbell B.T. (2008). Heterotic effects in topcrosses of modern and obsolete cotton cultivars. Crop Sci..

[7] Zhang X. (2016). Breeding potential of introgression lines developed from interspecific crossing between upland cotton (gossypium hirsutum) and Gossypium barbadense: heterosis, combining ability and genetic effects. PLoS One.

[8] Yu D. (2021). Molecular basis of heterosis and related breeding strategies reveal its importance in vegetable breeding. Hortic. Res..

[9] Fujimoto R. (2018). Recent research on the mechanism of heterosis is important for crop and vegetable breeding systems. Breed Sci..

[10] Labroo M.R. (2021). Heterosis and hybrid crop breeding: a multidisciplinary review. Front. Genet..

[11] Zhang T. (2023). Cotton heterosis and hybrid cultivar development. Theor. Appl. Genet..

[12] Vogel K.P., Mitchell R.B. (2008). Heterosis in switchgrass: biomass yield in swards. Crop Sci..

[13] Song X. (2011). Agronomic performance of F1, F2 and F3 hybrids between weedy rice and transgenic glufosinate‐resistant rice. Pest Manag. Sci..

[14] Zhang J.F., Abdelraheem A. (2017). Combining ability, heterosis, and genetic distance among nine elite American Pima cotton genotypes (Gossypium barbadense). Euphytica.

[15] Stella Galanopoulou-Sendouca a, b D.R. (1999). Performance of cotton F hybrids and its relation to the mean yield of advanced bulk generations. Eur. J. Agron..

[16] Smith E.N. (2023). Improving photosynthetic efficiency toward food security: strategies, advances, and perspectives. Mol. Plant.

[17] Evans J.R. (2013). Improving photosynthesis. Plant Physiol..

[18] Kautsky H., A H. (1931). Neue Versuche zur Kohlensäureassimilation. Naturwissenschaften.

[19] Guanter L. (2014). Global and time-resolved monitoring of crop photosynthesis with chlorophyll fluorescence. Proc. Natl. Acad. Sci. U. S. A..

[20] Gottardini E. (2014). Chlorophyll-related indicators are linked to visible ozone symptoms: evidence from a field study on native Viburnum lantana L. plants in northern Italy. Ecol. Indicat..

[21] Strasser R.J. (1995). Polyphasic chlorophyll a fluorescence transient in plants and cyanobacteria. Photochem. Photobiol..

[22] Luo H.-h. (2016). Combining gas exchange and chlorophyll a fluorescence measurements to analyze the photosynthetic activity of drip-irrigated cotton under different soil water deficits. J. Integr. Agric..

[23] Antunović Dunić J. (2023). Comparative analysis of primary photosynthetic reactions assessed by OJIP kinetics in three Brassica crops after drought and recovery. Appl. Sci..

[24] Srinivasarao C. (2016). Chlorophyll fluorescence induction kinetics and yield responses in rainfed crops with variable potassium nutrition in K deficient semi-arid alfisols. J. Photochem. Photobiol. B Biol..

[25] Kayoumu M. (2023). Phosphorus availability affects the photosynthesis and antioxidant system of contrasting low-P-tolerant cotton genotypes. Antioxidants.

[26] Hu W. (2018). Sub-optimal emergence temperature alters thermotolerance of thylakoid component processes in cotton seedlings. Environ. Exp. Bot..

[27] Snider J.L. (2018). OJIP-fluorescence parameters as rapid indicators of cotton (Gossypium hirsutum L.) seedling vigor under contrasting growth temperature regimes. Plant Physiol. Biochem..

[28] Virk G. (2021). Extreme temperatures affect seedling growth and photosynthetic performance of advanced cotton genotypes. Ind. Crop. Prod..

[29] Baroniya S.S. (2023). Intraspecific variation in photosynthetic efficiency in soybean (Glycine max L.) varieties towards solar ultraviolet radiations. Photosynthetica.

[30] Zharmukhamedov S.K. (2023). Probing the influence of novel organometallic copper(II) complexes on spinach PSII photochemistry using OJIP fluorescence transient measurements. Biomolecules.

[31] Morina F. (2023). Cadmium and Zn hyperaccumulation provide efficient constitutive defense against Turnip yellow mosaic virus infection in Noccaea caerulescens. Plant Sci..

[32] Gutie′rrez O.A. (2002). Genetic distance among selected cotton genotypes and its relationship with F2 performance. Crop Sci..

[33] William R., Meredith J. (1990). Yield and fiber-quality potential for second-generation cotton hybrids. Crop Sci..

[34] Anwar M. (2022). Inter-specific hybridization in cotton (gossypium hirsutum) for crop improvement. Agronomy.

[35] Hamid R. (2023). Dynamic roles of small RNAs and DNA methylation associated with heterosis in allotetraploid cotton (Gossypium hirsutum L.). BMC Plant Biol..

[36] Abdel-Aty M.S. (2023). Estimating the combining ability and genetic parameters for growth habit, yield, and fiber quality traits in some Egyptian cotton crosses. BMC Plant Biol..

[37] Alexandra J.Burgess (2022). Improving crop yield potential: underlying biological processes and future prospects. Food Energy Secur..

[38] Li G. (2021). Exploration of rice yield potential: decoding agronomic and physiological traits. The Crop Journal.

[39] Wasaya A. (2021). Evaluation of fourteen bread wheat (Triticum aestivum L.) genotypes by observing gas exchange parameters, relative water and chlorophyll content, and yield attributes under drought stress. Sustainability.

[40] Zhao F.-n. (2020). Quantifying key model parameters for wheat leaf gas exchange under different environmental conditions. J. Integr. Agric..

[41] Han S.-K. (2021). Stomatal lineage control by developmental program and environmental cues. Front. Plant Sci..

[42] Niinemets Ü. (2011). Evergreens favored by higher responsiveness to increased CO2. Trends Ecol. Evol..

[43] Lawson T., Vialet‐Chabrand S. (2018). Speedy stomata, photosynthesis and plant water use efficiency. New Phytol..

[44] Harrison E.L. (2019). The influence of stomatal morphology and distribution on photosynthetic gas exchange. Plant J..

[45] Carrera D.A. (2021). Distinct plastid fructose bisphosphate aldolases function in photosynthetic and non-photosynthetic metabolism in Arabidopsis. J. Exp. Bot..

[46] Lee B.-S. (2020). Overexpression of fructose-1,6-bisphosphate aldolase 1 enhances accumulation of fatty acids in Chlamydomonas reinhardtii. Algal Res..

[47] Arrivault S. (2019). Metabolite profiles reveal interspecific variation in operation of the Calvin-Benson cycle in both C4 and C3 plants. J. Exp. Bot..

[48] Carmo-Silva A.E., Salvucci M.E. (2013). The regulatory properties of Rubisco activase differ among species and affect photosynthetic induction during light transitions. Plant Physiol..

[49] Ivanov B.N. (2007). Diversity in forms and functions of carbonic anhydrase in terrestrial higher plants. Russ. J. Plant Physiol..

[50] Simkin A.J. (2017). Simultaneous stimulation of sedoheptulose 1,7-bisphosphatase, fructose 1,6-bisphophate aldolase and the photorespiratory glycine decarboxylase-H protein increases CO2 assimilation, vegetative biomass and seed yield in Arabidopsis. Plant Biotechnol. J..

[51] Matsumura H. (2020). Hybrid Rubisco with complete replacement of rice Rubisco small subunits by sorghum counterparts confers C4 plant-like high catalytic activity. Mol. Plant.

[52] Shomali Aida (2023). Artificial neural network (ANN)-based algorithms for high light stress phenotyping of tomato genotypes using chlorophyll fluorescence features. Plant Physiol. Biochem..

[53] Srivastava A., Strasser R.J. (1996). Stress and stress management of land plants during a regular day. J. Plant Physiol..

[54] Jiang Chuang-Dao (2003). Changes of donor and acceptor side in photosystem 2 complex induced by iron deficiency in attached soybean and maize leaves. Photosynthetica.

[55] Hermans C. (2003). Quality assessment of urban trees: a comparative study of physiological characterisation, airborne imaging and on site fluorescence monitoring by the OJIP-test. J. Plant Physiol..

[56] Philippus D. R. van Heerdena (2004). Reduction of dark chilling stress in N2-fixing soybean by nitrate as indicated by chlorophyll a fluorescence kinetics. Physiol. Plantarum.

[57] Kozuleva M.A., Ivanov B.N. (2023). Superoxide anion radical generation in photosynthetic electron transport chain. Biochemistry (Moscow).

[58] Li Li (2016). Carotenoids in Nature.

[59] Sun T. (2022). *Molecular Horticulture* 2.

[60] Delhom C.D. (2022). Cotton fibre elongation: a review. J. Text. Inst..

[61] Xu H. (2020). Assessment of seed yield and quality of winter oilseed rape using chlorophyll fluorescence parameters of pods. Transactions of the ASABE.

[62] Livak K.J., Schmittgen T.D. (2001). Analysis of relative gene expression data using real-time quantitative PCR and the 2−ΔΔCT method. Methods.

